# Supplementary material for: WordCommentsAnalyzer: A windows software tool for qualitative research
Source: F1000Res. 2018 Sep 4;7:536. Originally published 2018 May 3. [Version 2] doi: 10.12688/f1000research.14819.2 (PMC6137411; doi:10.12688/f1000research.14819.2)
Supplement: Supplementary file 3 [file f1000research-7-17614-s0002.tgz › b2a79496-4ada-47c4-9682-a99ce867836d.zip!/Eight Iranian Health Journals/Yafteh _54.docx]

© Yafteh  . This work is licensed under CC BY 4.0 (https://creativecommons.org/licenses/by/4.0/).

Volume & Issue: دوره 7 - شماره 3

Title: تاثیر ژل کلاژن بر روی رژنراسیون عصب سیاتیک در لوله پلی وینیلیدین فلوراید (مطالعه فانکشنال و هیستولوژی)

http://yafte.lums.ac.ir/article-1-1082-fa.html

مقدمه: محدودیت دسترسی به محل‌های دهنده مناسب برای پیوند عصب، ضرورت تحقیق جهت یافتن جانشین مناسب برای پیوند عصب را توجیه می‌ کند. هدف از این تحقیق مطالعه اثر مجرای پیزو الکتریک و ژل کلاژن بر روی رژنراسیون عصب بود. مواد و روش ها: این تحقیق یک مطالعه تجربی است که از 48 سر موش صحرایی نر (gr 250-200)استفاده شد. پس از قطع عصب سیاتیک، 1 سانتیمتر از عصب برداشته شد و شکاف حاصله توسط یکی از 4 روش زیر ترمیم شد (مجرای پلی وینیلیدین فلوراید یا PVDF با ژل کلاژن، اتوگرافت، شم و کنترل). همه حیوانات در روزهای 7، 21، 35، 49، 60 و 90 بعد از جراحی توسط تست‌های حسی،شاخص فعالیت عصب سیاتیک ((SFI و در نودمین روزبوسیله الکتروفیزیولوژی ارزیابی شدند. عصب سیاتیک در پایان هفته چهارم و دوازدهم بوسیله میکروسکوپ نوری و الکترونی مورد مطالعه قرار گرفت. نتیجه گیری: این مطالعه پیشنهاد می‌کند که با توجه به اثرات مثبت ژل کلاژن همراه با مجرای PVDF بر روی ترمیم عصب، استفاده از مجرای PVDF ممکن است جهت ترمیم ضایعات عصب محیطی مفید باشد. یافته ها: نود روز پس از جراحی، اختلاف معنی‌داری از لحاظ حسی در گروه‌های آزمایش مشاهده نشد. میانگین SFI در هفته نهم در گروه اتوگرافت نسبت به گروه ژل کلاژن+PVDF افزایش معنی داری داشت (01/0p<). اختلاف میانگین سرعت هدایت عصب حرکتی در گروه های آزمایش از لحاظ آماری معنی دار نبود. در پایان هفته چهارم پس از ترمیم، بافت عصب تشکیل شده داخل مجرای PVDF به شکل گرد بود و تعداد آکسون‌های میلین‌دار در گروه اتوگرافت بطور معنی داری بیشتر از گروه ژل کلاژن + PVDF بود (001/0p<). در پایان هفته دوازدهم پس از ترمیم، میانگین تعداد آکسون‌های میلین‌دار در گروه ژل کلاژن +PVDF نسبت به گروه اتوگرافت کمتر بود (01/0p<).

Title: فراوانی عوارض دیررس وازکتومی

http://yafte.lums.ac.ir/article-1-1083-fa.html

مقدمه: وازکتومی روشی نسبتا“ بی خطر و مؤثر در پیشگیری از بارداری است که به دلیل عدم نیاز به فن آوری پیچیده در خیلی از مناطق قابل اجرا است. با وجود این در کشور ما در برخی مناطق استقبال چندانی از آن نشده و در بسیاری از موارد این باور عمومی وجود دارد که وازکتومی باعث ایجاد ناتوانی جنسی در فرد مذکر می شود. هدف از انجام این مطالعه بررسی عوارض دیررس وازکتومی در افراد وازکتومی شده در شهرستان خرم آباد بود. مواد و روش ها: این مطالعه به صورت توصیفی مقطعی انجام شد و 110 نفر از مراجعه کنندگانی که در فاصله سالهای 81-78 در مرکز بهداشتی درمانی شماره 3 خرم آباد وازکتومی شده بودند، مورد بررسی قرارگرفتند. متغیرهای مورد مطالعه شامل میل جنسی، ناتوانی جنسی، گرانولوم، مشکل انزال،درد مفاصل، اختلال خواب، درد مزمن محل عمل، پشیمانی از عمل و ناموفقی عمل جراحی بود. اطلاعات مورد نیاز توسط پرسشنامه و معاینه افراد جمع آوری و با استفاده از آمار توصیفی مورد تجزیه وتحلیل قرار گرفت. یافته ها: بعد از ارزیابی های لازم روی 110نفر از مراجعه کنندگان، مشخص گردید که 60 درصد افراد، عدم تغییر در میل جنسی و 30 درصد از آنها افزایش میل جنسی داشتند. 5/85 درصد بدون ناتوانی جنسی، 5/85 درصد بدون گرانولوم و 9/90 درصد فاقد درد مفاصل بودند. 1/89 درصد شکایتی از اختلال انزال نداشتند. 5/95 درصد بدون اختلال خواب و 80 درصد درد مزمن محل عمل نداشتند. 3/97 درصد از انجام وازکتومی پشیمان نبودند. همچنین در هیچ موردی شکست عمل جراحی وجود نداشت. نتیجه گیری: در این مطالعه مشخص گردید که وازکتومی یک روش مؤثر و موفق در پیشگیری از بارداری است. دربیشتر موارد فاقد عوارضی از قبیل کاهش میل جنسی، ناتوانی جنسی، گرانولوم و... بود. در موارد اندکی که افراد وازکتومی شده از برخی عوارض شاکی بودند، نمی توان با قطعیت آن را صرفا ناشی از وازکتومی دانست و جهت بررسی بیشتر بایستی مطالعات تحلیلی و مورد- شاهد انجام گیرد.

Title: تأثیر مصرف طولانی مدت رانی‌تیدین بر ساختار هیستولوژیک بیضه رت بالغ

http://yafte.lums.ac.ir/article-1-1084-fa.html

مقدمه: باتوجه به اثر رانی تیدین در مهـار آنـزیـمهای Na+ K+ ATPase ، Ca++ ATPase وMg++ ATPase در بیضه‌ها ، افزایش سطح پرولاکتین ، اختلال در متابولیسم استروژن درکبد و گزارشاتی مبنی بر ژینکوماستی تأثیر مصرف طولانی مدت رانی تیدین بر بیضه مورد مطالعه قرار گرفت. مواد و روش ها: در این مطالعه تجربی 40 رت نر بالغ دو ماهه آلبینو به چهار گروه 10 تایی (یک گروه کنترل و سه گروه آزمایشی 1، 2 و 3) تقسیم شدند. حیوانات گروه آزمایشـی 1 ، 2 و 3 برای مدت دو ماه به ترتیب تحت تجویز دهانی رانـی‌تیدیـن با دوزهای 10 ، 70 و400 mg/kg/day قرار گرفتند. بیضـه راست برداشته و در محلول بوئن ثابت گردید. بعد از پردازش بافتی و برش گیری ، برشها با روشهای H&E ، پاس و تریکروم رنگ‌آمیزی و مورد مطالعه کیفی و مرفومتریک قرار گرفتند.متغیرهای کمی با تست توکی و p<0.05 به کمک نرم افزار spss12 ارزیابی شد. نتیجه گیری: رانی تیدین دردوز اپتی مم و دراز مدت ضایعه ای درساختار طبیعی بیضه ایجاد نکرده واثر معنی داری بر تغییر قطر و ضخامت پوشش لوله های سمینی فر نشان نمی دهد. یافته ها: رانی تیدین دردوز اپتی مم(g/kg/day10) و دراز مدت ضایعه بافتی درساختار طبیعی بیضه ایجاد نکرده و بر تغییر قطر و ضخامت پوشش لوله های سمینی فرنسبت به گروه کنترل تأثیر معنی داری نشان نداد. رانی تیدین در دوزهای بالا (70 و400 mg/kg/day) موجب ایجاد شکاف در پوشش لوله ها ، حفره دار شدن لوله ها ، آتروفی لوله ها و کاهش معنی دار ضخامت پوشش لوله های سمینی فر شد (05/0p<)؛ ولی موجب تغییر قطر لوله ها شد.

Title: بررسی میزان باز بودن عروق کرونر در درمان با هیبرکیناز (Heberkinase)

http://yafte.lums.ac.ir/article-1-1085-fa.html

مقدمه: تزریق ترمبولیتیک ها هنوز شایع ترین روش درمانی انفارکتوس حاد قلبی(MI) در کشور مااست. با توجه به گزارشاتی که در مورد تأثیر ناکافی نوعی از این دارو که با نام تجارتی Heberkinase در سال های اخیر در کشور ما مصرف می شود دریافت شده است، مطالعه زیر به منظور بررسی میزان باز بودن عروق کرونر درگیر در MI به دنبال تزریق Heberkinase طراحی و انجام شد. مواد و روش ها: در این مطالعه گذشته نگر110 بیمار مبتلا به انفارکتوس حاد میوکارد که در طی 12 ساعت اول بعداز شروع علائم, این ترمبولیتیک را دریافت نموده بودندو در طی 20 روز اول بعد از درمان آنژیوگرافی کرونرشده بودند، انتخاب شدند. جریان خون کرونر با درجه بندی 2و3 در مقیاس TIMI با تنگی زیر 95 % به عنوان رگ باز (Patent) در نظر گرفته شد. یافته ها با کسر تخلیه ای عوامل خطر و اطلاعات دمو گرافیک بیماران و نیز با مطالعات دیگر در این زمینه مقایسه شد. یافته ها: در 49 % بیماران ماده ترمبولیتیک طی 2 ساعت، در 39% در بین ساعت 6-2 و 12%، 12-6 ساعت بعد از شروع علائم تزریق شده بود. 73 بیمار در 10 روز اول و 37 بیمار بین روز دهم تا بیستم آنژیوگرافی شده بودند و متوسط زمان انجام آنژیوگرافی 6/4 + 5/8 روز بود. متوسط میزان باز بودن رگ انفارکتوس در زمان انجام آنژیوگرافی 55 % بود. میزان باز بودن با محل MI و رگ درگیر ارتباط معنی داری نداشت. در مقایسه انجام شده کسر تخلیه ای در گروه رگ باز به طرز معنی داری از گروه دیگر بالاتر بود (47 % در مقابل 42 %) (03/0p<).نتیجه گیری: در مقایسه با سایر مطالعات مشابه میزان باز بودن مشاهده شده در بیماران ما درحدود 15 % کمتر از مطالعات مشابه بود و نیز در مقایسه با آماری که در دوران قبل از شروع ترمبو لیتیک درمانی در مورد میزان باز بودن خود به خود عروق داده شده است تفاوت واضحی در بیماران ما دیده نشد.

Title: سرواپیدمیولوژی لیشمانیوز احشایی (عفونت انسانی) با روش آگلوتیناسیون مستقیم

http://yafte.lums.ac.ir/article-1-1086-fa.html

مقدمه: لیشمانیوز احشایی یکی از بیماریهای عفونی انگلی است که از نظر بهداشتی اهمیت زیادی دارد. این مطالعه در منطقه میانکوه شرقی شهرستان پلدخترازاستان لرستان با هدف تعیین سرواپیدمیولوژی لیشمانیوز احشایی (عفونت انسانی) در سال 83-1382 انجام گرفته است. مواد و روش ها: مطالعه به شکل توصیفی ـ مقطعی بوده و روش نمونه برداری به شکل تصادفی چند مرحله ای و گروههای هدف بچه های زیر 12 سال ساکن در منطقه و 10% از بزرگسالان بود. در مجموع 530 نمونه خون از افراد مذکور تهیه شد که بعد از تهیه پلاسما با روش سرولوژی آگلوتیناسیون مستقیم (DAT)به منظور تشخیص آنتی بادی ضد لیشمانیایی مورد آزمایش قرار گرفتند. در تست DAT برای نمونه های انسانی تیتر های 1:3200 و بالاتر مثبت وتیترهای 1:1600 مشکوک درنظر گرفته شد. یافته ها: با استفاده از روشDAT،6نفر(26/1%)دارای تیتر1:3200 و بالاتر ازنظر سرولوژی مثبت بودند و 1 نفر (21/0%) باتیتر1:1600 به عنوان تیتر مشکوک و 6نفردارای تیترهای 1:800 مشاهده شد. این مطالعه در تمام روستاهای میانکوه شرقی انجام گرفت که موارد مثبت سرمی به ترتیب از روستاهای لتوند(امورد)،دارصافه (2مورد)بن جو(2 مورد)و عشایر کوچنده (1مورد)بودند. در سایر روستاهاهیچ موردی از بیماری مشاهده نگردید. نتیجه گیری: نتایج نشان می دهد که لیشمانیوزاحشایی یک بیماری اسپورادیک درمنطقه میانکوه شرقی پلدختر می باشد. این بررسی لزوم مطالعات تکمیلی جهت شناسایی ناقلین قطعی و مخازن احتمالی دیگر در منطقه را پیشنهادمی کند.

Title: مقایسه شروع اثر آتراکوریوم ـ آلفنتانیل و ساکسی نیل کولین وریدی در بیماران تحت روش اینتوباسیون سریع

http://yafte.lums.ac.ir/article-1-1089-fa.html

مقدمه: یکی از پر مصرف ترین داروهای بی هوشی، ساکسی نیل کولین می باشد که در ابتدای بی هوشی جهت شلی عضلانی و لوله گذاری، مورد استفاده قرار می گیرد. هدف از انجام این مطالعه مقایسه اثر آتراکوریوم + آلفنتانیل با ساکسی نیل کولین در بیماران تحت اینتوباسیون سریع، می باشد. مواد و روش ها: در این کارآزمایی بالینی، 100 بیمار نیازمند به لوله گذاری تراشه به صورت تصادفی به دو گروه 50 نفری تقسیم شدند. در یک گروه از بیماران از روش معمول ساکسی نیل کولین (کنترل) و در گروه آزمایش آتراکوریوم + آلفنتانیل، سپس تزریق نسدونال که آلترناتیوی برای روش R.S.I می‌باشد، استفاده شد و اختلاف بین این دو گروه را با توجه به وضعیت لوله‌گذاری تراشه و بررسی وضعیت شلی فک، حرکات وکال کوردها و زور زدن ارزیابی شد. داده ها با استفاده از آزمون کای دو و نرم افزار SPSS نسخه 1/9 تجزیه و تحلیل شد. نتیجه گیری: روش آتراکوریوم + آلفنتانیل می‌تواند به عنوان یک جایگزین برای روش استاندارد در مواردی که ساکسی‌نیل‌کولین منع مصرف دارد، باشد. برای حرکات خفیف وکال کوردها و زور زدن خفیف در روش آتراکوریوم + آلفنتانیل، پیشنهاد می شود افزایش کمی در دوز آتراکوریوم می‌تواند مشکلات فوق الذکر را حل نماید. یافته ها: تجزیه و تحلیل داده ها نشان داد زور زدن در افراد گروه آلفنتانیل بعلاوه آتراکوریوم در 62% موارد وجود نداشت و در 38% موارد به صورت خفیف وجود داشت؛ ولی در گروه دریافت کننده ساکسی نیل کولین زور زدن وجود نداشت (0001/0p<) در مورد شلی فک تفاوتی وجود نداشت. حرکت طنابهای صوتی در گروه دریافت کننده آلفنتانیل وجود داشت در حالیکه در گروه دریافت کننده ساکسی نیل کولین مشاهده نگردید.

Title: میزان حمایت اجتماعی خانواده از افراد HIV مثبت و مبتلایان به ایدز

http://yafte.lums.ac.ir/article-1-1088-fa.html

مقدمه: بیماری ایدز یک مشکل بزرگ است که بر روی تمام جنبه های زندگی فرد تاثیر می گذرد. وحشت طردشدن از اجتماع یا خانواده و دوستان موجب مخفی ساختن بیماری و گسترش آلودگی می شود. خانواده بهترین مکان برای حمایت از افراد آلوده و مبتلا به ایدز است و حمایت خانواده می تواند موجب ارتقا، کیفیت زندگی آنها و پیشگیری ازا نتقا ل عفونت شود. این مطا لعه با هدف تعیین میزان حمایت اجتماعی خانواده از افرادHIV مثبت ومبتلایان به ایدز شهر خرم آباد در سال 1383انجام شد. مواد و روش ها: پژوهش حاضر یک مطالعه توصیفی – مقطعی است که در آن کلیه افرادHIV مثبت و مبتلایان به ایدز شهر خرم آباد (147 نفر) که ابتلا و یا آلودگی آنها تا اردیبهشت سال 83 تائید شده و خانواده هایشان از بیماریشان اطلاع داشتند، مورد بررسی قرار گرفتند. ابزار گرد آوری داده ها شامل یک پرسشنامه 2 قسمتی حاوی 36 سئوال بود که قسمت اول شامل16 سؤال مربوط به مشخصات فردی واحدهای مورد پژوهش و قسمت دوم شامل 20 سئوال مربوط به حمایت اجتماعی خانواده بر اساس پرسشنامه استاندارد میزان حمایت اجتماعی خانواده بود. کلیه پرسشنامه ها پس از مراجعه به منازل واحد های مورد پژوهش تکمیل شد. یافته ها: نتایج پژوهش نشان داد که اکثریت واحدهای مورد پژوهش (5/56%) از حمایت اجتماعی ضعیف خانواده برخوردار بودند. میزان حمایت اجتماعی خانواده افراد متاهل بیشتر از افراد مجرد بود که این اختلاف معنی دار بود (01/0=p). افراد متاهلی که حمایت اجتماعی بیشتری داشتند نسبت به افراد متاهل کم حمایت در روابط زناشوئی خود بیشتر از کاندوم استفاده می کردند که این اختلاف نیز معنی دار بود (04/0=P). افرادی که حمایت اجتماعی بیشتری داشتند در مواقعی که امکان آلوده کردن دیگران وجود داشت بیشتر مشکل خود را به آنها گوشزد می کردند که این اختلاف معنی دار بود (05/0p=) در این پژوهش ارتباط معنی داری بین حمایت اجتماعی و احساس مسئولیت این بیماران در پیشگیری از ابتلا سایرین و گوشزد کردن مشکل خود به دیگران در موا قعی که امکان ابتلا سایرین وجود داشت، بدست آمد (00/0=p). نتیجه گیری: بر اساس نتایج پژوهش افرادی که حمایت اجتماعی خانواده از آنها بیشتر بود، به پیشگیری از ابتلا سایرافراد اهمیت بیشتری می دادند و در شرایطی که امکان آلودگی آنها وجود داشت بیشتر مشکل خود را به آنها گوشزد می کردند. با توجه به اینکه ارضاء انسان از حمایت اجتماعی خانواده موجب جلوگیری از درماندگی روانی ودرنتیجه کارآمدی اجتماعی بهتر و احساس مسئولیت بیشتر در قبال سلامت جامعه می شود و ]ون در این بررسی اغلب واحدهای مورد پژوهش از حمایت اجتماعی ضعیفی برخوردار بودند لذا توصیه می شود که با آگاه کردن و آموزش خانواده مبتلایان؛ حمایت اجتماعی از این بیماران را ارتقا دهیم تا امکان آلودگی را در جامعه کاهش دهیم.

Title: تأثیر غلظت‌های تحت مهاری عصاره الکلی بابونه آلمانی برروی فعالیت آنزیم کاتالاز استافیلوکوکوس اورئوس

http://yafte.lums.ac.ir/article-1-1090-fa.html

مقدمه: بابونه آلمانی گیاهی است داروئی که خواص درمانی متعددی دارد.به دلیل وجود مقدار زیادی ترپنوئید - بیزابولول در عصاره این گیاه خواص ضد میکروبی آن قابل توجه است. استافیلوکوکوس اورئوس باکتری بیماریزائی است که نقش مهمی در پزشکی و بهداشت مواد غذائی دارد. از آنجائیکه کاتالاز به عنوان آنزیم تجزیه کنندهٌ آب اکسیژنه نقش مهم و حیاتی در فیزیولوژی, تعیین هویت و بیماریزائی بسیاری از باکتری ها از جمله استافیلوکوکوس اورئوس دارد، لذا بررسی تاثیر غلظت های تحت مهاری عصاره الکلی گیاه بابونهٌ آلمانی بر روی فعالیت این آنزیم هدف از اجرای تحقیق می باشد.مواد و روش ها: در این تحقیق بنیادی ـ کاربردی گیاه بابونهٌ آلمانی ازباغ مرکز تحقیقات جهاد کشاورزی استان تهران تهیه و به وسیلهٌ اتانول 85 درجه عصاره گیری شد. حداقل غلظت های مهاری و کشندگی عصاره بر روی سویهٌ استاندارد استافیلوکوکوس اورئوس به روش رقت در براث تعیین وسپس تاثیر غلظت های تحت مهاری عصاره بر فعالیت آنزیم کاتالاز باکتری به صورت نیمه کمی و کمی با استفاده از روش تیتراسیون با پرمنگنات پتاسیم ارزیابی شد. یافته ها: رقت های 1:32 و 1:16 به ترتیب به عنوان حداقل غلظت های مهاری و کشندگی عصاره تعیین شد. عصاره در غلظت های تحت مهاری 1:64 و 1:128 توانست به ترتیب 8/82% و 8/32% فعالیت آنزیم کاتالاز باکتری را نسبت به شاهد کاهش دهد.0 نتیجه گیری: افت شدید و چشم‌گیر فعالیت آنزیم مذکور در غلظت‌های کمتر از مهار کنندگی عصاره, نسبت به شاهد بدون عصاره احتمالاً نمایانگر تأثیر بر روی یک یا چند مرحله از فرایندهای تولید این آنزیم در سطح ژنوم، رونویسی, ترجمه، انتقال و.... می باشد؛لذا لازم است در این زمینه مطالعات تکمیلی بیشتری انجام گیرد. این آنزیم‌ می‌تواند به عنوان هدفی جدید در طراحی و دستیابی به برخی از مواد ضدباکتریایی باشد.

Title: مقایسه میزان βHCG سرم در خانم های باردار مبتلا به پره اکلامپسی نسبت به خانمهای باردار با فشار خون طبیعی

http://yafte.lums.ac.ir/article-1-1091-fa.html

مقدمه: پره اکلامپسی به عنوان یک علت مهم پریناتال مرتالیتی و مربیدیتی در جهان باقی مانده است و همراه با خونریزی و عفونت، تریاد عوامل مرگبار مادران را تشکیل می‌دهند. فاکتورهای متعددی جهت تشخیص زودرس این عارضه و یا تعیین شدت و وخامت این بیماری مشخص شده است. از آنجایی که اختلال تروفوبلاستی یکی از اتیولوژی‌های مطرح شده در این بیماری می‌باشد بر آن شدیم که میزان βHCG سرم را که منعکس‌کننده میزان فعالیت تروفوبلاست می‌باشد در خانم‌های باردار با فشار خون طبیعی و پره اکلامپسی خفیف و شدید تعیین نماییم. مواد و روش ها: این مطالعه مقایسه‌ای بر روی 75 خانم حامله مراجعه کننده به بیمارستان عسلیان که از نظر سنی و گراویتی تقریباً همسان بودند انجام شد. خانم‌های حامله در سه گروه 25 نفری با فشار خون طبیعی پره اکلامپسی خفیف و پره اکلامپسی شدید قرار گرفتند. سطح سرمی βHCG به روش ELISA در هر سه گروه اندازه‌گیری و با یکدیگر مقایسه شد. یافته ها: میزان βHCG در خانم‌های حامله با فشار خون طبیعی 2123 ± 18572 واحد در لیتر بود و میزان آن در خانم‌های حامله با پره اکلامپسی خفیف 6558 ± 23962 و در خانمهای حامله مبتلا به پره اکلامپسی شدید 4634 ± 59220 بوده است. بر اساس آزمون آماری کروسکال-والیس سطح βHCG در گروه پره اکلامپسی شدید با دو گروه دیگر تفاوت آماری معنی‌دای را نشان می‌دهد (001/0pv <) اما بین دو گروه با فشار خون طبیعی و پره اکلامپسی خفیف تفاوت معنی‌دار وجود نداشت. نتیجه گیری: سطح βHCG در خانم‌های پره اکلامپسی شدید بیشتر از خانم‌های با فشار خون طبیعی و یا مبتلا به پره اکلامپسی خفیف بوده است. بنابراین افزایش βHCG می‌تواند شدت وخامت بیماری را نشان دهد اما در مورد تشخیص زودرس بیماری ارزش چندانی ندارد.

Title: بررسی نقش نارسائی کلیه در بروز اختلالات چربیهای خون

http://yafte.lums.ac.ir/article-1-1092-fa.html

مقدمه: حوادث قلبی عروقی از مهمترین عوامل مرگ ومیر در جوامع انسانی است. از آنجا که نارسائی کلیه یکی از علل شناخته شده در بروز دیس لیپیدمی است، نقش آن درایجاد دیس لیپیدمی و میزان تاثیر آن بر شیوع این اختلال در جامعه مورد بررسی قرار گرفت. مواد و روش ها: دراین مطالعه اپیدمیولوژیک مقطعی 10136 نفرازافراد بیست ساله و بالاتر از ساکنین منطقه 13 تهران بطور تصادفی انتخاب شد و اطلاعات کامل پزشکی، وضعیت تن‌سنجی (آنتروپومتریک)،میزان فعالیت بدنی و سطح سرمی تری‌گلیسرید، کلسترول تامLDL-C, HDL-C,، FBS,BS(2h post 75 gr orall Glucose)، Cr، AlK.P، T4 و TSH اندازه گیری و آزمایش کامل ادرار انجام شد. براساس اطلاعات فوق افراد مورد مطالعه به دو گروه: سالم و دیس‌لیپیدمیک تقسیم شدند. افراد دیس لیپیدمیک خود به دو گروه دیس‌لیپیدمی اولیه و ثانویه تفکیک شدند وبا در نظر گرفتن سایر علل ثانویه دیس لیپیدمی و استفاده از آزمایش رگرسیون، ارتباط بین نارسائی کلیه و دیس لیپیدمی مورد تجزیه و تحلیل قرار گرفت. در این تحقیق از روش‌های آماری X2، ANOVA، T.test، رگرسیون خطی و لوجستیک بهره گرفته شده است. یافته ها: جمعیت مورد مطالعه که واجد تمامی اطلاعات لازم جهت بررسی بودند جمعاً 9632 نفر شامل 5619زن و 4013 مرد بودند. 8/65%زنان و3/72% مردان مبتلا به نوعی از دیس‌لیپیدمی بودند که شیوع کلی آن 5/68% جامعه را شامل می‌شود. در جامعه مورد تحقیق کلا 13 نفر مبتلا به نارسائی کلیه بودند که تمامی آنان مبتلا به حداقل یک نوع اختلال لیپید بودند که شایعترین نوع اختلال به ترتیب: هیپرکلسترولمی، کاهش HDLC،افزایش LDLC و درنهایت هیپر تری گلیسرید بود (05/0p<). نتیجه گیری: جامعه شهری شرق تهران که یک نمونه فراگیر از کل جمعیت شهری است همچون سایر جوامع شهری از شیوع بالای دیس‌لیپیدمی رنج می‌برد و درصد بالای جمعیت مبتلا به نوع ثانویه دیس ‌لیپیدمی است. نارسائی کلیه یکی از این عوامل زمینه ای است که توجه ویژه ای را طلب می کند.
